# Inhibition of BCR/ABL Protein Expression by miR-203 Sensitizes for Imatinib Mesylate

**DOI:** 10.1371/journal.pone.0061858

**Published:** 2013-04-16

**Authors:** Yajuan Li, Ying Yuan, Kun Tao, Xin Wang, Qing Xiao, Zhenglan Huang, Liang Zhong, Weixi Cao, Jianping Wen, Wenli Feng

**Affiliations:** 1 Key Laboratory of Laboratory Medical Diagnostics and Department of Clinical Hematology Designated by the Ministry of Education, Chongqing Medical University, Chongqing, China; 2 Department of General Surgery, The First Affiliated Hospital of Chongqing Medical University, Chongqing, China; 3 Department of Immunology, Chongqing Medical University, Chongqing, China; 4 Department of Hematology, The First Affiliated Hospital of Chongqing Medical University, Chongqing, China; 5 Department of Pathology and Molecular Medicine, McMaster University, Ontario, Canada; Mayo Clinic, United States of America

## Abstract

Selective inhibition of BCR/ABL expression by RNA interference has been demonstrated as an effective strategy in CML treatment and a reversal to imatinib resistance. microRNAs (miRNAs) are small regulatory RNAs involved in post-transcriptional gene regulation. miR-203 is supposed to directly regulate ABL and BCR/ABL expression, however, the role of miR-203 in imatinib-resistant cells is not clear. Here, we report that overexpression of miR-203 in BaF3-BCR/ABL cells with T315I mutant inhibited cell growth and colony formation ability. Furthermore, miR-203 increased sensitivity to imatinib in BaF3-BCR/ABL^T315I^ cells, thereby antagonizing the main mechanism of resistance to imatinib.

## Introduction

The reciprocal chromosomal translocation t(9; 22) results in the highly stable, constitutively active tyrosine kinase BCR/ABL, which inhibits apoptosis and triggers malignant transformation of chronic myelogenous leukemia (CML). Treatment with imatinib mesylate, a potent ABL-specific tyrosine kinase inhibitor (TKI), has shown remarkable clinical activity in a majority of CML patients in chronic phase, but in fewer patients in accelerated and blast phase [1]. However, resistance to imatinib can emerge due to point mutations or BCR/ABL amplification [2]. While most BCR/ABL mutant forms are sensitive to the second-generation TKIs (dasatinib and nilotinib), the BCR/ABL T315I mutation remains completely refractory to imatinib, as well as to these new agents [3]. Therefore, the investigation of alternative treatment for CML is still of clinical significance.

It has been proved that silencing BCR/ABL by small interfering RNA (siRNA) selectively inhibits BCR/ABL- dependent cell growth [4] and increases sensitivity to imatinib in BCR/ABL positive cells [5], [6]. Combining BCR/ABL siRNA with imatinib or nilotinib had additional effects on induction of apoptosis and inhibition of proliferation of wild-type and mutated BCR/ABL cells [5]. These results further indicate that circumventing the resistance of the BCR/ABL kinase to imatinib by directly interfering with its mRNA is another attractive strategy currently been studied.

microRNAs (miRNAs) are noncoding RNAs involved in post-transcriptional gene regulation [7]. They regulate gene expression by inducing translational inhibition or cleavage of target mRNAs through binding to a target site in the 3′- untranslated region (3′UTR) [8]. Emerging evidence has demonstrated that altered expression of miRNA has been implicated in various cancers and it can function as tumor suppressors or oncogenes, depends on the target they regulate [8]. miR-203, which is silenced in T cell malignancies, is supposed to directly regulate ABL and BCR/ABL expression [9]. However, the detailed role of miR-203 in BCR/ABL positive cells with point mutations, especially T315I mutation, is not clear.

In the present study, we examined the expression of miR-203 in CML patient samples and found that miR-203 was significantly downregulated in CML patients. This study provides experimental evidence that enforced expression of miR-203 was able to inhibit proliferation of BCR/ABL positive cells with T315I mutation. Moreover, interference BCR/ABL expression with miR-203 restored the sensitivity to imatinib in cells expressing the imatinib-resistant BCR/ABL kinase domain mutant T315I.

## Materials and Methods

### Cell culture

The murine interleukin 3 (IL-3)-dependent pre-B lymphoid cell line BaF3 was obtained from BoPei Biotech Co. Ltd (Chongqing, China). Imatinib-resistant BaF3-BCR/ABL mutant cell line BaF3-BCR/ABL^T315I^ was established from BaF3 cells by stable transfection with plasmids expressing mutated BCR/ABL^T315I^ transcripts. Cells were grown in RPMI 1640 medium (Gibco) with 10% fetal calf serum (FCS) in a humidified incubator at 37°C in 5% CO_2_.

### Patient samples

Bone marrow samples from 8 CML patients (Table 1) collected at diagnosis were used. Leukemic cells were collected using Ficoll gradient (Tianjin Haoyang Biological Manufacture Co. Ltd., China). Mature monocytes or granulocytes from five healthy volunteers were isolated from peripheral blood. Total RNA was extracted using RNAiso Plus (Takara, China) and reverse transcribed into cDNA for miRNA expression detection by real-time RT-PCR. The study was approved by the human ethics committee of Chongqing Medical University. A written informed consent was obtained from all patients and controls in this study.

**Table 1:** Clinical information of the CML patients.

Sample	Age (y)	Sex	Phase at diagnosis
P1	35	Male	Accelerated
P2	44	Male	Accelerated
P3	64	Female	Blast crisis
P4	48	Male	Chronic
P5	56	Male	Chronic
P6	42	Male	Chronic
P7	36	Male	Chronic
P8	50	Male	Blast crisis

### Generation of stable cell lines

To overexpress miR-203, lentivirus encoding miR-203 were prepared by Genepharma (Shanghai, China). BaF3-BCR/ABL^T315I^ cells were infected with virus encoding miR-203 or negative control (NC) and selected with puromycin for two weeks. Polybrene (6 µg/ml) was added to enhance the infection. The cell line stably expressing miR-203 or NC was named as BaF3-BCR/ABL^T315I^-miR203 or BaF3-BCR/ABL^T315I^ -NC, respectively.

### Real-time RT-PCR

Total RNA was extracted in RNAiso Plus (Takara, China) and reverse transcribed into cDNA using a PrimeScript® RT reagent Kit with gDNA Eraser (Takara, China). Real time quantitative PCR (qPCR) was performed using a SYBR premix Ex Taq™ kit (Takara, China) on the Mini Opticon Real-time PCR detection System (Bio-Rad, USA). For analysis of miRNA expression, reverse transcription and PCR were carried out using a Bulge-Loop™ miRNA qPCR Primer Set for miR-203 and U6 snRNA (RiboBio, China) according to the manufacturer's instructions. The relative expression of miRNA was calculated using the comparative 2^−ΔΔCt^ method and was normalized using U6 snRNA.

### Cell proliferation and colony formation assay

BaF3-BCR/ABL^T315I^, BaF3-BCR/ABL^T315I^ -NC and BaF3-BCR/ABL^T315I^ -miR203 cells were plated into 96-well flat-bottomed microtiter plates at a concentration of 1×10^4^ cells per well. At different time points (0 h, 24 h, 48 h, 72 h), cell proliferation assays using the MTT method were performed as described previously [10]. Cells were exposed to different concentrations of imatinib (Novartis) with different time (0 h, 24 h, 48 h, 72 h) and cell survival was measured by MTT assay.

For colony formation assay, cells were washed with PBS and plated in methylcellulose (200 cells/well). Colonies were counted 8 days later using an inverted microscope (Olympus, Japan). All analyses were performed in triplicates.

### Cell cycle analysis

To perform cell cycle analysis, cells were washed and fixed in 70% ethanol at 4°C overnight. Fixed cells were stained with propidium iodide and subjected to flow cytometry.

### Western blot

Cell lysis, SDS-PAGE, and immunoblotting were done as described previously [10]. Antibodies against c-ABL, Bax, STAT5 and phosphorylated-STAT5, PARP, caspase 3 were from Cell Signaling Technology (Beverly, MA). Antibodies against p21, p27, cyclin D1, Bcl-2, and β-actin were from Santa Cruz Biotechnology (Santa Cruz, CA). Antibodies against Rb1 were from Abzoom Biolabs (Dallas, USA). Anti-mouse and anti-rabbit HRP-conjugated secondary antibody were from Santa Cruz Biotechnology (Santa Cruz, CA). Bands were visualized using the ECL system (Millipore, USA) and were semi-quantified using Gel-Pro Analyzer. β-actin was used as loading control.

### Statistical analysis

Data are expressed as the mean ± SD. All the data was repeated at least thrice. The difference between the control and experimental test was analyzed by Student's *t*-test. The value of P<0.05 was considered statistically significant.

## Results

### Expression profile of miR-203 in CML patient samples

**Figure 1:**
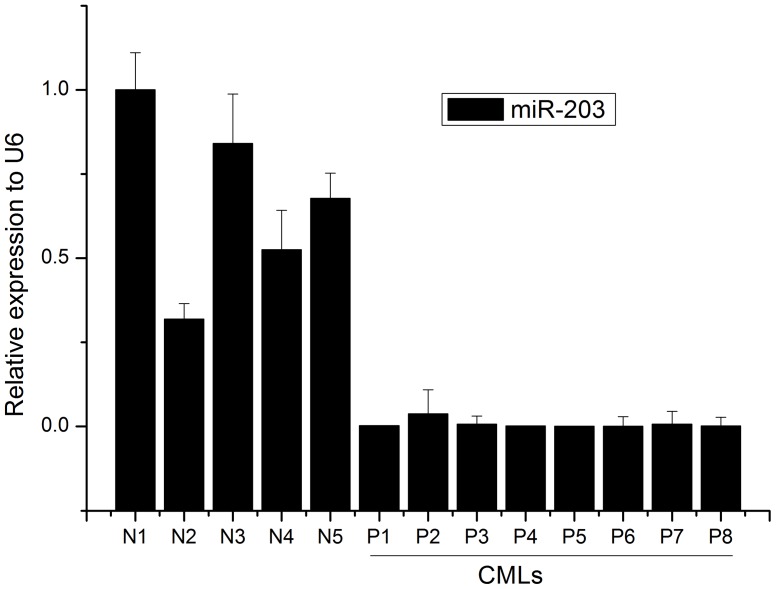
The expression profile of miR-203 in CML. Quantification of miR-203 was measured by stem-loop real-time PCR in human CMLs (P1–P8) using purified white cells. N1–N5 indicate samples getting from healthy volunteers using as controls. Histograms represent mean ± SD.

In order to confirm whether miR-203 was involved in the pathogenesis of CML, we assessed the miR-203 expression level in CML patient samples. As shown in Fig. 1 and Table 1, miR-203 was significantly downregulated as compared with healthy volunteers.

### Generation of stable cell lines expressing miR-203

**Figure 2:**
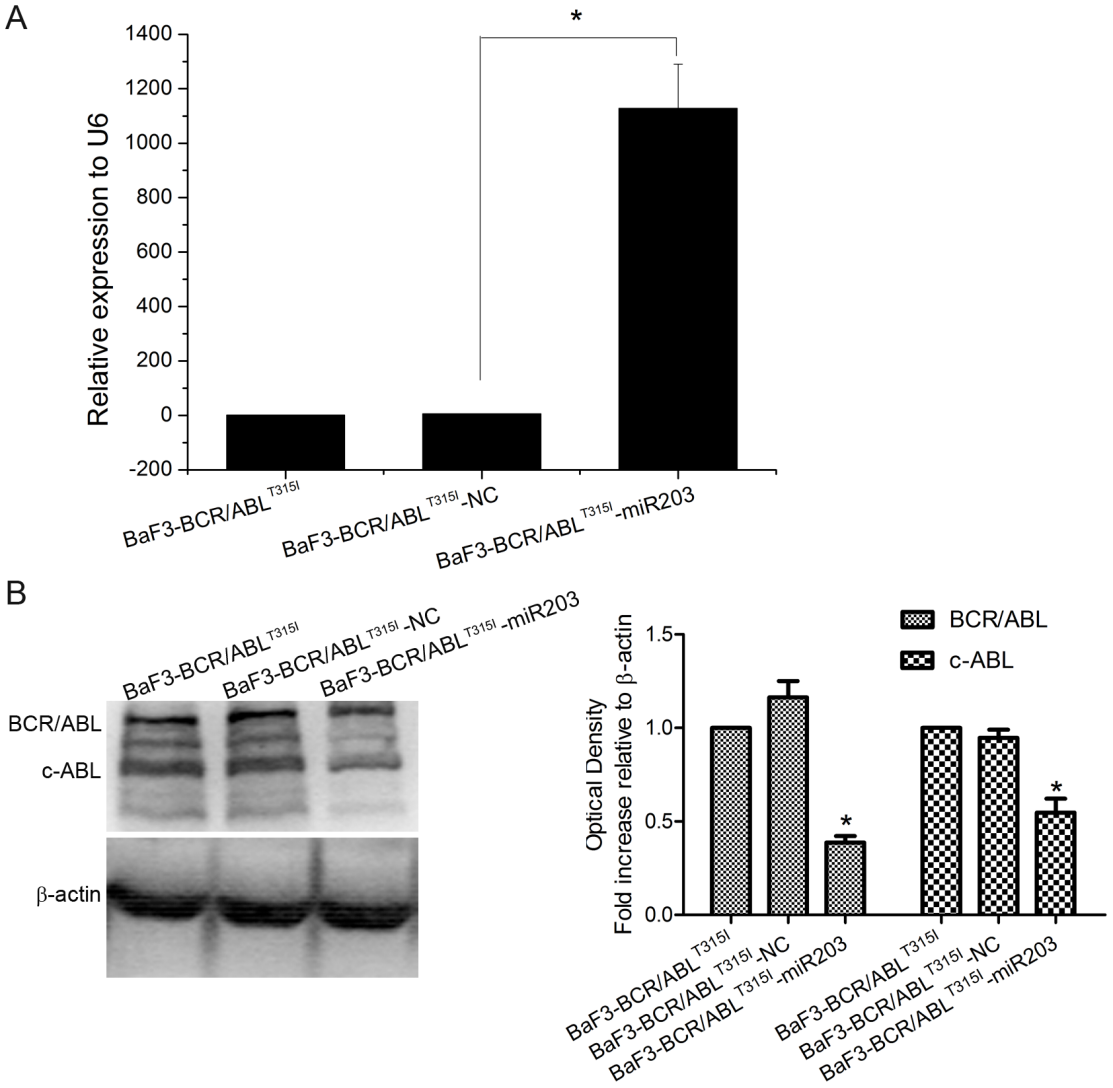
Characterization of BaF3-BCR/ABL^T315I^-miR203 stable cell line. BaF3-BCR/ABL^T315I^ cells infected with NC or miR-203 lentivirus were selected with puromycin for two weeks. Cells were harvested and total RNA was isolated and analyzed for the expression of miR-203 (A), BCR/ABL protein (B) using qPCR and Western blot, respectively. One representive blot out of three is presented and β-actin is used as internal control. Densiometric analysis of BCR/ABL and c-ABL correlated to β-actin level is presented as fold increase compared with untreated BaF3-BCR/ABL^T315I^ cells. * P<0.05 versus control groups.

To investigate the role of miR-203 in imatinib resistant BaF3-BCR/ABL^T315I^ cells, we prepared lentiviral vectors to overexpress miR-203. BaF3-BCR/ABL^T315I^ cells were infected with lentivirus encoding miR-203 or scrambled sequence used as negative control (NC) and stable cell lines BaF3-BCR/ABL^T315I^-NC and BaF3-BCR/ABL^T315I^-miR203 were generated. Increased expression of miR-203 (Fig. 2A) and decreased protein expression of c-ABL and BCR/ABL (Fig. 2B) were detected in BaF3-BCR/ABL^T315I^-miR203, but not in BaF3-BCR/ABL^T315I^-NC cells.

### Effects of miR-203 overexpression on BaF3-BCR/ABL^T315I^ cell proliferation

**Figure 3:**
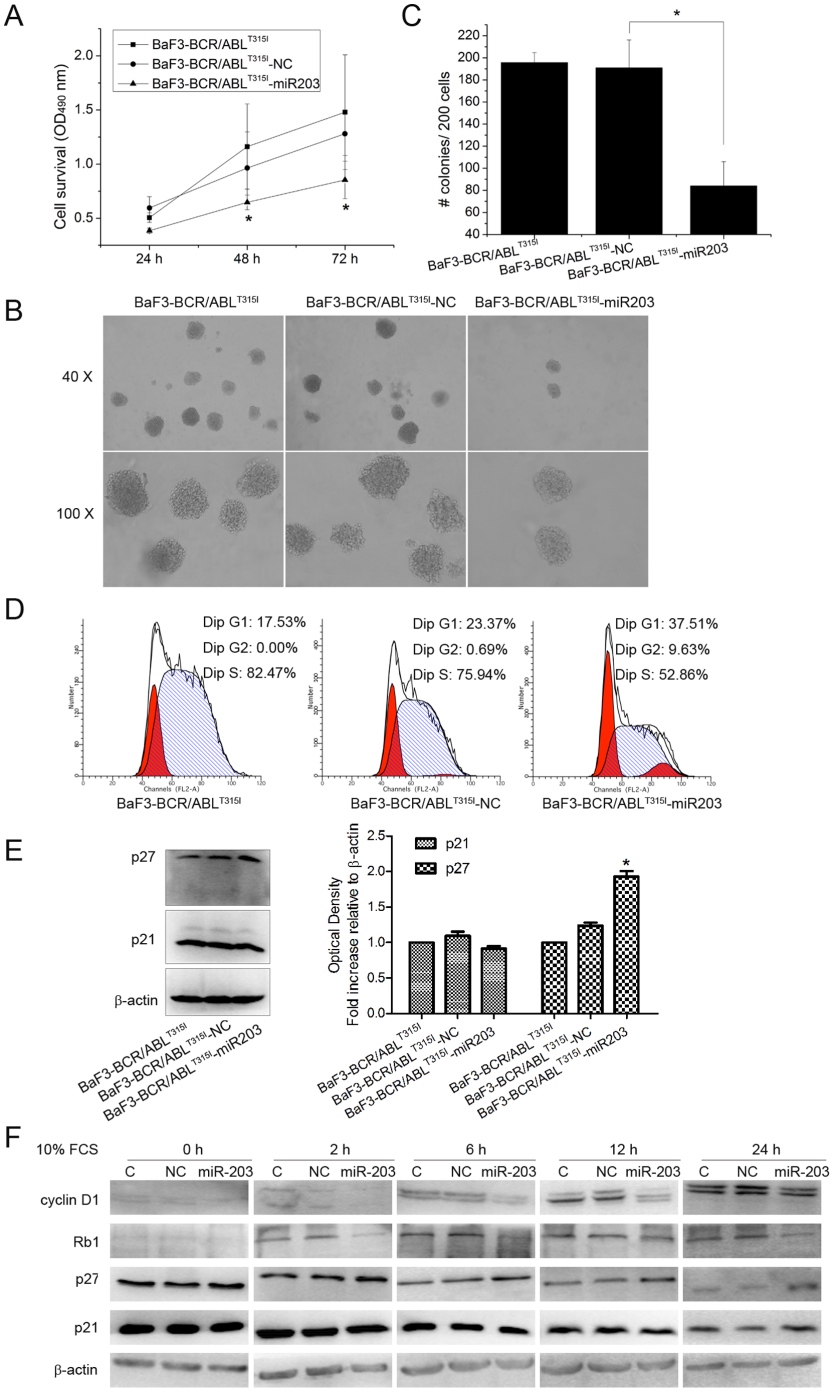
Overexpression of miR-203 inhibits the growth of BaF3-BCR/ABL^T315I^ cells. (A) Growth of BaF3-BCR/ABL^T315I^, BaF3-BCR/ABL^T315I^ -NC and BaF3-BCR/ABL^T315I^-miR203 cells was compared using MTT assay. (B) Cells were cultured in methylcellulose for 8 days and colonies containing ≥50 cells in each well were counted. (C) Histogram and statistics showing the number of colonies per 200 plated cells. (D) Cells without synchronized were collected for cell cycle analysis. The percentages of cells in the G1, G2, and S phases were evaluated by flow cytometry and BaF3-BCR/ABL^T315I^-miR203 cells were arrested in the G1 phase. (E) The protein levels of G1 cell cycle regulatory proteins p21 and p27 were evaluated using Western blot with lysates from cells without synchronized. β-actin was used as internal control. Densiometric analysis of p21 and p27 correlated to β-actin level is presented as fold increase compared with untreated BaF3-BCR/ABL^T315I^ cells. * P<0.05 versus control groups. (F) Cells were left hungry for 48 h without serum (0 h). After serum starvation, the synchronized cells were incubated with 10% FCS for 2 h, 6 h, 12 h, and 24 h. Then, these cells were harvested for Western blot to determine the level of p21, p27, cyclin D1 and Rb1. β-actin was used as internal control. C, NC and miR-203 indicate lysates from control cell line BaF3-BCR/ABL^T315I^, BaF3-BCR/ABL^T315I^ -NC and BaF3-BCR/ABL^T315I^-miR203, respectively.

To explore the possible function of miR-203 on the growth of BaF3-BCR/ABL^T315I^ cells, the dynamics of cell growth were determined by MTT assay. As shown in Fig. 3A, enforced expression of miR-203 significantly decreased the growth rate of BaF3-BCR/ABL^T315I^ cells in a time-dependent manner. Furthermore, the average colony number of BaF3-BCR/ABL^T315I^-miR203 decreased compared with BaF3-BCR/ABL^T315I^ or BaF3-BCR/ABL^T315I^-NC cells (Fig. 3B and C). To further explore the cause for the decrease in cell viability, the profile of cell cycle distribution in BaF3-BCR/ABL^T315I^ cells was analyzed by flow cytometry (Fig. 3D). Then, cells were cultured in serum-free medium to synchronize the cells at the G1 phase. As shown in Table 2, at 48 h after serum starvation, about 53% of the BaF3-BCR/ABL^T315I^ cells were in the G1 phase regardless of the miR-203 status. When the cells were cultured in the medium containing 10% FCS for 6 h, 37.57% of the BaF3-BCR/ABL^T315I^ cells were in the G1 phase while 43.56% of the BaF3-BCR/ABL^T315I^-miR203 cells were in the G1 phase. The difference was more remarkable at 12 h, when 26.70% of the BaF3-BCR/ABL^T315I^ cells and 40.20% of the BaF3-BCR/ABL^T315I^-miR203 cells were in the G1 phase.

**Table 2:** The effects of miR-203 on BaF3-BCR/ABL^T315I^ cell cycle progression^a^.

Cell type	Length of culture (h)	% of cells in indicated phase		
		G1	G2	S
BaF3-BCR/ABL^T315I^	0	52.83	12.08	35.09
BaF3-BCR/ABL^T315I^-NC		52.94	13.90	33.16
BaF3-BCR/ABL^T315I^-miR203		53.23	13.64	33.13
BaF3-BCR/ABL^T315I^	2	44.43	14.58	40.99
BaF3-BCR/ABL^T315I^-NC		46.20	14.31	39.50
BaF3-BCR/ABL^T315I^-miR203		47.81	14.01	38.18
BaF3-BCR/ABL^T315I^	6	37.57	21.62	40.82
BaF3-BCR/ABL^T315I^-NC		37.44	18.29	44.27
BaF3-BCR/ABL^T315I^-miR203		43.56	15.59	40.85
BaF3-BCR/ABL^T315I^	12	26.70	23.33	49.97
BaF3-BCR/ABL^T315I^-NC		27.14	22.09	50.77
BaF3-BCR/ABL^T315I^-miR203		40.20	20.31	39.50
BaF3-BCR/ABL^T315I^	24	19.15	3.90	76.96
BaF3-BCR/ABL^T315I^-NC		22.32	2.08	75.60
BaF3-BCR/ABL^T315I^-miR203		31.85	4.41	63.74

a Cells were incubated for 48 h in serum-free medium to synchronize cells in the G1 phase at 0 h. After that, cells were incubated in the medium with 10% FCS and collected at 2 h, 6 h, 12 h, and 24 h. The percentages of cells in G1, G2, and S phases were evaluated by flow cytometry.

Then, we examined the effect of miR-203 on cell cycle-regulatory molecules operative in the G1 phase. We assessed the effect of miR-203 on the induction of p21 p27, cyclin D1, and Rb1, which were reported to regulate the entry of cells from the G1 phase to S phase. Western blot results revealed that enforced miR-203 expression resulted in a significant induction of p27 and downregulation of cyclin D1 and Rb1, whereas the expression of p21 was not changed among these cells (Fig. 3E and F).

### Combined treatment with miR-203 and imatinib results in increased cellular responses

**Figure 4:**
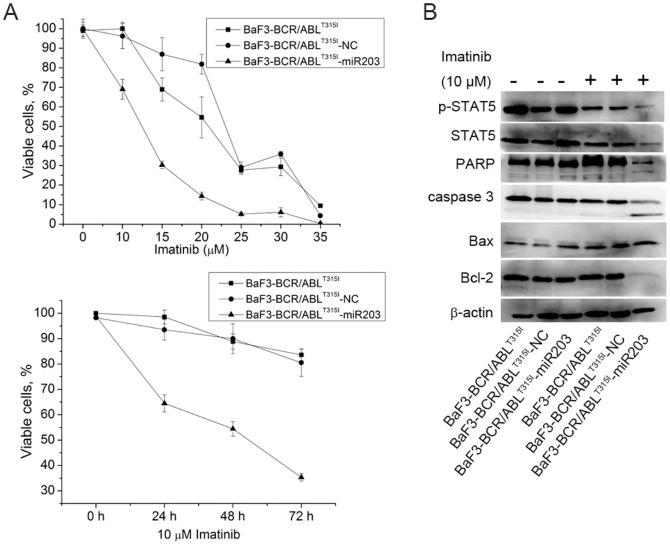
Combined treatment with miR-203 and imatinib results in increased cellular responses. (A) BaF3-BCR/ABL^T315I^, BaF3-BCR/ABL^T315I^ -NC and BaF3-BCR/ABL^T315I^-miR203 cells were treated with different concentrations of imatinib (0–35 µM) and the viability of cells was determined by MTT assay (above). Moreover, cells were treated with 10 µM imatinib for different time periods and the viability of cells was determined by MTT assay (below). (B) Lysates from BaF3-BCR/ABL^T315I^, BaF3-BCR/ABL^T315I^ -NC and BaF3-BCR/ABL^T315I^-miR203 cells treated with 10 µM imatinib were examined for Bax, Bcl-2, caspase 3, PARP, STAT5 and p-STAT5 expression by Western blot. β-actin was used as internal control.

We further determined whether the inhibition of BCR/ABL by miR-203 is sufficient to overcome resistance to imatinib. BaF3-BCR/ABL^T315I^ and cells overexpressed of miR-203 were treated with different concentrations of imatinib and the viability of cells was determined by MTT assay. BaF3-BCR/ABL^T315I^-NC or BaF3-BCR/ABL^T315I^ cells retained higher viability than BaF3-BCR/ABL^T315I^-miR203 cells at all drug doses (Fig. 4A). Furthermore, when treated with 10 µM imatinib for different time periods, the viability of BaF3-BCR/ABL^T315I^-miR203 cells was decreased in a time-dependent manner (Fig. 4A). We also observed more significant downregulated expression of p-STAT5 in BaF3-BCR/ABL^T315I^-miR203 cells (Fig. 4B). To confirm that enforced expression of miR-203 and imatinib were inducing apoptosis, caspase 3 and its substrate PARP were measured. BaF3-BCR/ABL^T315I^-miR203 cells had higher caspase 3 and PARP activation than control cells (Fig. 4B). Moreover, treatment of BaF3-BCR/ABL^T315I^-miR203 cells with imatinib was associated with increasing amounts of the proapoptotic factor Bax and decreasing levels of the antiapoptotic protein Bcl-2, both regulating apoptotic cell death by mitochondrial-mediated pathway (Fig. 4B). Therefore, these results demonstrated that BaF3-BCR/ABL^T315I^-miR203 cells were more sensitive to imatinib induced cell death than BaF3-BCR/ABL^T315I^ or BaF3-BCR/ABL^T315I^-NC cells.

## Discussion

In the current study, we characterized the role of miR-203 in imatinib-resistant cell lines. We examined the expression of miR-203 in CML patients by qPCR and the results demonstrated that miR-203 was significantly reduced in CML patients' bone marrow samples as compared with healthy volunteers' (Fig. 1), which confirmed that miR-203 was involved in the pathogenesis of CML. We further assessed the combined treatment effects of miR-203 and imatinib on imatinib-resistant cell lines and determined that miR-203 can served as a novel target for CML treatment.

Previous studies revealed that CML is characterized by aberrant BCR/ABL tyrosine kinase activities [11]. To date, much efforts have been focused on targeting aberrantly activated BCR/ABL using tyrosine kinase inhibitors, such as imatinib and perhaps the even more effective second-generation drug nilotinib. However, many patients develop resistance to these drugs due to acquired BCR/ABL mutations or amplification [2]. Selective silencing of BCR/ABL expression by RNA interference is proved to induce BCR/ABL positive cell death [12]. In addition, transfection of siRNA targeting BCR/ABL increased the sensitivity of imatinib-sensitive and imatinib-resistant CML cell lines to imatinib [6]. Koldehoff et al. showed that silencing of BCR/ABL by siRNA also restored the sensitivity to imatinib and nilotinib of BCR/ABL positive cells with T315I and H396P mutants [5]. Thus, RNA interference technology provides an alternative strategy for CML treatment and a reversal to tyrosine kinase inhibitors resistance.

miRNAs, functions as an siRNA, can regulate protein synthesis post-transcriptionally by base-pairing to target mRNAs [13]. Growing evidence indicates that miRNAs can serve as oncogenes or tumor suppressors in tumorigenesis [14] and correction of specific miRNA alterations using miRNA mimics or antagomirs can normalize the gene regulatory network and further reverse the phenotype in cancer cells [15]. miR-203, which is downregulated in many cancers, regulates multiple cellular processes associated with proliferation, differentiation, metastasis and apoptosis [16], [17], [18], [19]. Restoration of miR-203 expression reduces ABL and BCR/ABL levels through binding its 3′UTR and inhibits CML cell lines K562 and KCL-22 proliferation [9]. To determine the effect of miR-203 in imatinib-resistant cell line, stable cell line overexpressed of miR-203 was constructed and characterized by qPCR and Western blot (Fig. 2). Enforced expression of miR-203 significantly suppressed cell growth and colony formation ability of BaF3-BCR/ABL^T315I^ (Fig. 3A–C). These results are further supported by the data from cell cycle analysis which show that overexpression of miR-203 significantly increased the percentage of cells in G1 phase mainly through inducing the expression of the cell cycle-dependent kinase inhibitors p27 and decreasing the expression of cyclin D1 and Rb1 (Fig. 3D–F).

To assess whether miR-203 sensitizes BaF3-BCR/ABL^T315I^ to imatinib, cells were treated with different concentrations of imatinib and BaF3-BCR/ABL^T315I^-miR203 cells were more sensitive to imatinib treatment as compared with negative control cells in a time-dependent manner (Fig. 4A). We further investigated the potential mechanisms involved in miR-203 and imatinib induced apoptosis in BaF3-BCR/ABL^T315I^ cells. As shown in Fig. 4B, higher activation of caspase 3 and PARP were observed in BaF3-BCR/ABL^T315I^-miR203 cells. Apoptosis induced by caspase activation is regulated by the balance between Bcl-2 and Bax [20]. Indeed, we found that the ratio of Bax/Bcl-2 was increased by miR-203, thus rendering cells more prone to induction of apoptosis.

In summary, we demonstrated that enforced expression of miR-203 effectively suppressed growth of BaF3-BCR/ABL cells with T315I mutant. Moreover, additive effects of miR-203 with imatinib result in anti-proliferative and enhanced proapoptotic effects in BaF3-BCR/ABL^T315I^ cells.
